# Clinical Significance of Tumor Infiltrating Lymphocytes and Modulation by Neoadjuvant Chemotherapy in Colorectal Cancer Liver Metastases

**DOI:** 10.3390/cancers18183042

**Published:** 2026-09-19

**Authors:** Yasaman Rezaie, Kerryan Ashley, Janie Zhang, Thejal Srikumar, Jassim DiPalermo, Kurt A. Schalper, Michael Cecchini

**Affiliations:** 1 Department of Internal Medicine, Yale School of Medicine, Yale University, New Haven, CT 06520, USA; 2 Department of Pathology, Yale School of Medicine, Yale University, New Haven, CT 06520, USA; 3 Department of Internal Medicine, University of Pittsburgh (UPMC), Pittsburgh, PA 15213, USA; zhangjy2@upmc.edu

**Keywords:** colorectal cancer (CRC), colorectal cancer liver metastasis (CRCLM), tissue infiltrating lymphocytes (TILs), quantitative immunofluorescence (qIF), neoadjuvant chemotherapy, CD8^+^ T-cell, immune microenvironment

## Abstract

**Background**: Colorectal cancer liver metastases (CRCLMs) are associated with treatment resistance, and poor survival. We aimed to measure the differences in the immune composition of primary colorectal tumors and CRCLMs to identify prognostic and potentially actionable differences in the tumor microenvironment (TME). **Methods**: We created two independent cohorts of patients that had surgical resection of primary colorectal tumor and CRCLM resection at the Yale Cancer Center, comprising of 84 and 95 cases arranged in tissue microarray (TMA) format. Using multiplex quantitative immunofluorescence (QIF) we measured CD4^+^, CD8^+^, and FOXP3^+^ immune cells across spatially-resolved compartments within the tumor samples. We then assessed the association of clinicopathologic variables and treatment-specific outcomes with immune cell composition. **Results**: CRCLMs showed significantly higher CD8^+^ effector T-cells and significantly lower FOXP3^+^ regulatory T-cell infiltration than primary tumors. In addition, high CD8^+^ T-cell infiltration within the cancer cell compartment of CRCLMs was prominently associated with longer overall survival in both cohorts (Cohort1: HR = 0.38, *p* = 0.032; Cohort2: HR = 0.23, *p* = 0.046). Neoadjuvant chemotherapy (NAC) was associated with a significant increase in CD8^+^ T-cells across all tumor tissue regions in CRCLMs (*p* < 0.01 in both cohorts). However, NAC was not associated with improved three-year progression-free survival in either cohort (Cohort1: HR = 0.75, *p* = 0.43; Cohort2: HR = 0.97, *p* = 0.93). **Conclusions**: The TME of CRCLMs is characterized by reduced Tregs and increased TILs in NAC-treated patients. Furthermore, increased CD8^+^ infiltration in the tumoral compartment within CRCLMs is associated with improved survival. Our results describe local adaptive anti-tumor immune responses seen in CRCLM and underscore the need for novel therapeutic strategies that leverage TME alterations.

## Simple Summary

Colorectal cancer frequently spreads to the liver, where tumors resist immunotherapy through poorly understood mechanisms. Using quantitative multiplexed immunofluorescence in two independent patient cohorts, we measured CD4^+^, CD8^+^, and FOXP3^+^ regulatory T-cells in matched primary tumors and liver metastases. We found that high CD8^+^ T-cell infiltration in the tumor compartment of liver metastases was independently associated with significantly better overall survival. Neoadjuvant chemotherapy increased T-cell infiltration but did not improve survival outcomes, suggesting chemotherapy alone cannot restore effective anti-tumor immunity in this setting. These findings identify CD8^+^ T-cells as a clinically meaningful biomarker in colorectal liver metastases and highlight the need for targeted strategies to enhance immune function at metastatic sites.

## 1. Introduction

Colorectal cancer remains a leading cause of worldwide cancer mortality, and long-term outcomes for patients with stage IV disease remains poor. The liver is the most common site of dissemination and colorectal cancer liver metastasis (CRCLM) account for 80% of all metastases. Approximately 20% of all patients diagnosed with colorectal cancer have CRCLM at the time of diagnosis and 50% will develop CRCLM during the disease course [1,2,3].

Chemotherapy remains the mainstay of treatment for most patients with mismatch repair-proficient (pMMR) CRCLM. While immunotherapy has transformed outcomes across many malignancies and provides clear clinical benefit in mismatch repair-deficient (dMMR) CRC [4,5], its efficacy in pMMR disease has been limited. Cytotoxic chemotherapy can induce an inflammatory response within tumors, remodel the tumor microenvironment, and alter antigenic expression [6], thereby providing a biological rationale for combining immune checkpoint inhibitors with chemotherapy. Nevertheless, the results of such combinations in pMMR CRC have been mixed [7,8], highlighting the unmet need for more effective immunomodulatory strategies and precision approaches tailored to the unique immunosuppressive niche of CRCLM.

Clinically, patients with liver metastatic cancer develop resistance to systemic immunotherapy across multiple cancer types, suggesting that local responses can have global immune effects in patients with disseminated disease [9]. In CRCLM, the development of immunomodulatory approaches has been limited by well-documented resistance to immune checkpoint inhibitors (ICIs) in these tumors [10,11]. Hypotheses for drivers of tumor immune evasion in CLCLM have centered on the unique immunosuppressive tumor microenvironment (TME). This immune composition is characterized by abundant M2-like immunosuppressive macrophages, myeloid derived suppressor cells (MDSCs) and regulatory T-cells (Tregs) that support a premetastatic niche and tumor progression [12,13,14]. For example, metastatic tumor cells can recruit and reprogram hepatic macrophages through secreted cytokines and modify cell to cell interactions to foster premetastatic niche formation and metastatic progression [12,13,14]. However, the liver has mechanisms to provoke robust local innate immune activation through mechanisms such as Toll-like receptor signaling, molecular danger patterns (alarmins), and inflammasome activation [15]. The balance between proinflammatory and anti-inflammatory pathways and the functional diversity of the immune cells involved, including macrophages and T-cells, can determine the outcome of immune response including immune tolerance or anti-tumor immunity [15,16].

The liver TME is dynamic and can be modulated by anti-cancer therapies such as neoadjuvant chemotherapy (NAC) [12]. Previous studies support that NAC prior to liver metastectomy can induce a transient recruitment of CD8^+^ cytotoxic T-cells, producing a more inflamed, T-cell-rich TME landscape [12,17]. The role and adaptations of tumor-infiltrating lymphocytes (TILs) and other aspects of adaptive immunity in CRCLMs versus primary colonic tumors are complex and poorly understood. The possible impact of NAC on immune cells beyond effector TILs and their impact on patient outcomes remain uncertain.

To assess the T-cell composition and clinical significance of TILs in patients with CRCLM we compared major TIL populations in matched primary tumors and CRCLMs from patients in two independent cohorts. We employed analytically validated quantitative immunofluorescence (QIF) panels combined with high-resolution image-based analysis allowing for quantitative and spatially resolved output and integration with patient and tumor-level data. Our findings reveal key aspects of the immune landscape of CRCLMs with possible biomarker implications and could also inform the development of future treatment approaches aiming to enhance antitumoral immunity in the liver.

## 2. Methods

### 2.1. Samples and Cohorts

We established two independent retrospective cohorts including tumor samples from resected primary adenocarcinoma of the colon/rectum and CRCLM from patients seen at Yale-New Haven Hospital between 2000 and 2024. A total of 161 and 146 formalin-fixed, paraffin-embedded (FFPE) primary or metastatic tumors were retrospectively reviewed from 84 and 95 patients with stage IV colorectal cancer who underwent primary CRC tumor resection and metastasectomy in Cohort1 (discovery cohort) and Cohort2 (validation cohort), respectively. Patients included in the cohorts had different stages at diagnoses and were either diagnosed with stage IV disease or progressed to stage IV after being diagnosed with an earlier stage. All cases were reviewed by a pathologist using hematoxylin and eosin (H&E) stained slides, and tumor cores for tissue microarray (TMA) construction were selected from the most representative tumor regions. Each tumor was sampled in 2–4 cores from distinct areas and assembled in tissue microarray (TMA) blocks. Clinicopathologic data was extracted from the electronic medical record.

### 2.2. Multiplexed Immunofluorescence Staining

The multiplex immunofluorescence staining protocol used for the sample analysis has been previously reported and described in detail [18,19,20]. Analytical performance of the QIF assay was carried out by staining multiple positive and negative control tissues, as well as single-plex to multiplex concordance analysis. Briefly, TMA slides were deparaffinized and rehydrated through a graded alcohol series, followed by antigen retrieval using EDTA buffer (Sigma-Aldrich, St. Louis, MO, USA, Cat# 03690, pH 8.0) in a pressure-boiling module (PT Module, Lab Vision, Fremont, CA, USA) at 97 °C for 20 min. Slides were then incubated at room temperature with methanol containing 2.5% hydrogen peroxide for 20 min to block endogenous peroxidase activity, followed by overnight incubation at 4 °C in 0.3% BSA in 0.05% Tween to block nonspecific binding.

The sequential multiplexed immunofluorescence protocol was performed using isotype-specific primary antibodies to detect epithelial tumor cells (Cytokeratin Alexa-488 conjugated, clone AE1/AE3, eBioscience, San Diego, CA, USA), helper T-cells (CD4 IgG, 1:100, clone SP35, SpringBio, Piscataway, NJ, USA), cytotoxic T-cells (CD8 IgG1k, 1:250, clone C8/144B, Dako, Carpinteria, CA, USA), and regulatory T-cells (FOXP3 IgG, 1:25, clone D2W8E, Cell Signaling Technology, Danvers, MA, USA), followed by secondary antibodies and fluorescent detection reagents. Secondary antibodies and fluorophores included anti-mouse Envision (Agilent Technologies, Santa Clara, CA, USA, Cat# K4001), anti-rabbit Envision (Agilent Technologies, Cat# K4003), goat anti-rabbit (Abcam, Cambridge, UK, 1:500, Cat# GR3391208), TSA Cyanine 5 (Akoya Biosciences, Marlborough, MA, USA, Cat# SAT705A001EA), TSA Plus Cyanine 3 (Akoya Biosciences, Cat# NEL744001KT), and a biotinylated tyramide/streptavidin-Alexa750 conjugate (Thermo Fisher Scientific, Waltham, MA, USA, Cat# S21384). Nuclei were stained using 4′,6-diamidino-2-phenylindole (DAPI). Residual horseradish peroxidase activity between staining cycles was quenched by treating slides twice for 7 min at room temperature with a solution containing 0.136 mg benzoic hydrazide and 50 µL hydrogen peroxide in PBS. Reproducibility of the QIF was confirmed by analyzing serial sections from an index TMA, which included both positive and negative controls, across multiple time points.

### 2.3. Tissue Fluorescence Measurement and Scoring

TMA slides were scanned using a multispectral Vectra Polaris imaging system (Akoya Biosciences) at 20× magnification with fixed exposure time applied across all samples. Image analysis was performed using the Automated Quantitative Analysis (AQUA^®^) method, which is based on a pixel-level fluorescence co-localization algorithm and allows for objective and sensitive quantification of biomarkers within user-defined tissue compartments [19,21,22]. QIF scores for each marker in the total tissue area (e.g., total compartment), cytokeratin-positive (CK^+^) cancer cell compartment (e.g., tumor compartment), and in the surrounding non-tumor stromal cells (e.g., stromal compartment) were calculated by dividing the target fluorescence signal intensity by the area of DAPI^+^, CK^+^, and/or CK^−^ image pixel regions. Scores were normalized to enable cross-sample comparison.

### 2.4. Statistical Analysis

QIF signal intensities across markers and compartments were analyzed using mean fluorescence scores and compared using the non-parametric Mann–Whitney U test. Patient characteristics were assessed using Student’s *t*-test for continuous variables and the χ^2^ test for categorical variables. Survival analyses were conducted using Kaplan–Meier estimates with statistical significance determined by the log-rank test. Case stratification for survival analysis was based on optimal cut-points associated with each marker, as determined using a custom informatic platform named SCRIBE^®^ (manuscript under review). The cut-points generated using survival analysis for marker stratification in the first cohort were then applied to the second cohort (70th percentile for CD4 and CD8 and 50th percentile for FOXP3). Statistical analyses were performed using JMP Pro v17 and GraphPad Prism v10.4.1. A two-tailed *p*-value ≤ 0.05 was considered as statistically significant.

## 3. Results

**Figure 1:**
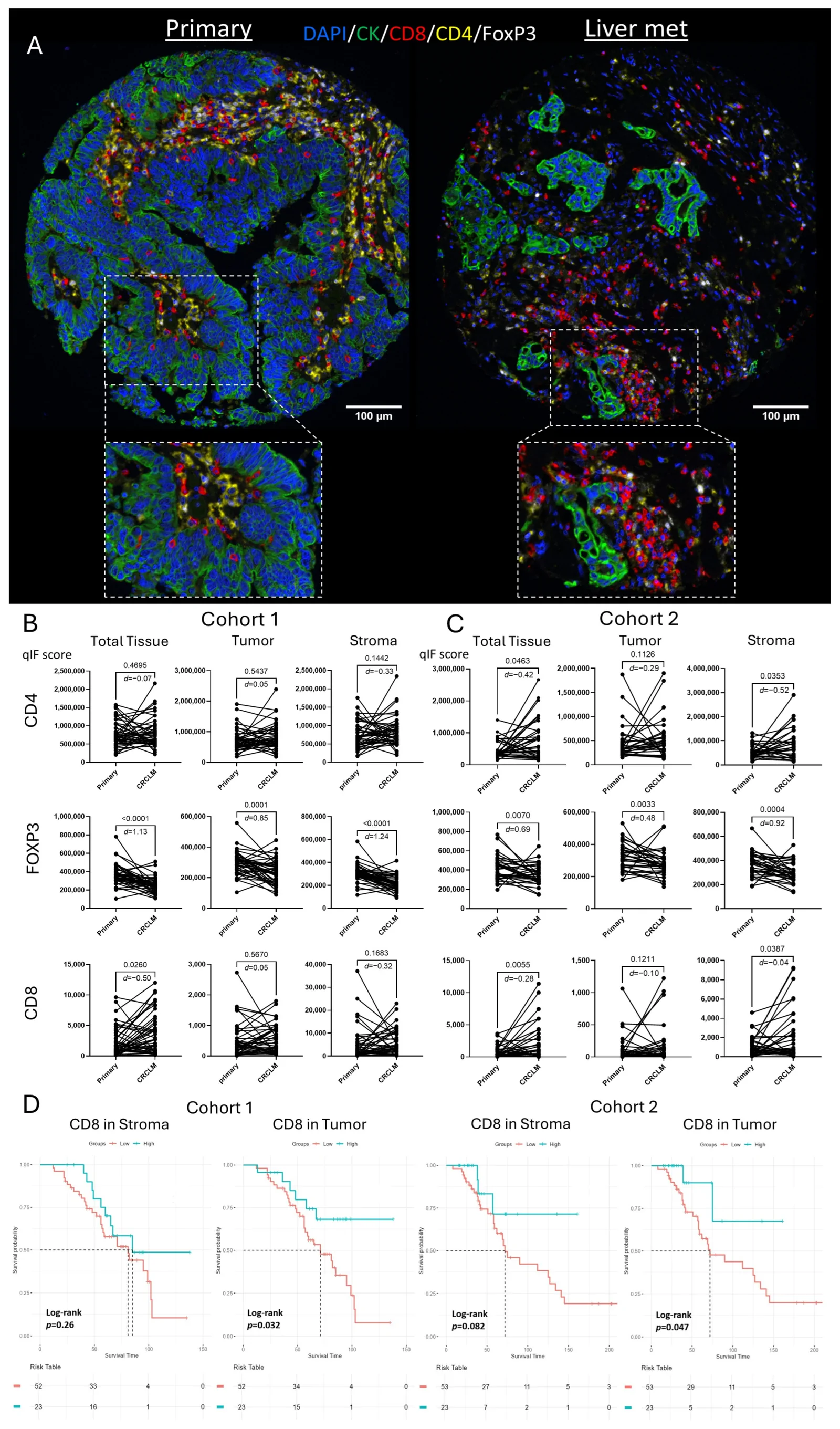
The TILs composition and survival outcomes. (**A**). Representative immunofluorescence caption from CRC cohorts demonstrating TIL infiltration in a primary tumor and CRCLM tissue core. (**B**,**C**). Paired analysis of TILs infiltration in primary and CRCLM cores from Cohort1 and Cohort2 respectively.; Each dot represents the mean tissue qIF score in one patient; lines connect paired samples from the same patient. *p* values shown above the line, Cohen’s *d* values shown below (**D**). Kaplan–Meier curves demonstrating significant overall survival benefits with higher CD8^+^ TIL infiltration (blue line) in tumor compartments of CRCLM cores in both cohorts. Dotted line indicates median survival.

The detailed clinicopathological characteristics of the two patient cohorts are shown in Table 1. No significant differences were seen in patient’s age, sex, race, stage, sidedness of the primary colorectal tumor, grade, and mean follow-up duration. Representative images of stained TILs in a primary tumor and the corresponding CRCLM are shown in Figure 1A. A representative example of tissue segmentation can be seen in Supplementary Figure S1. The expression of immune cell type markers exhibited a continuous spectrum across samples (Supplementary Figure S2A). Analysis of the marker levels across different tumor tissue compartments showed higher levels of TILs in the stromal vs. tumor compartment, with CD8 showing the most dramatic decline in the tumor compartment of both cohorts (Supplementary Figure S2A). Pairwise analysis revealed positive correlations between the levels of CD4, CD8, and FOXP3 signal, although no correlation reached significance (Supplementary Figure S2B). As shown in Figure 1B,C, comparative analysis of markers measured in the stroma and total tissue areas of matched CRCLM and primary tumor tissue from each patient showed higher CD4^+^ TILs in CRCLM in both cohorts, although it reached statistical significance only in Cohort2 (*p* = 0.04 and 0.03, Cohen’s *d* = −0.42 and −0.52 in Cohort2 total tissue area and stroma, respectively). Total CD8^+^ TILs levels were significantly higher in CRCLM whole tissue compartments from both cohorts (*p* = 0.02 and 0.005, Cohen’s *d* = −0.50 and −0.28 in Cohort1 and Cohort2, respectively). FOXP3^+^ TILs were significantly lower in CRCLM in all tissue compartments of both cohorts (*p* < 0.01 in all compartments).

**Figure 2:**
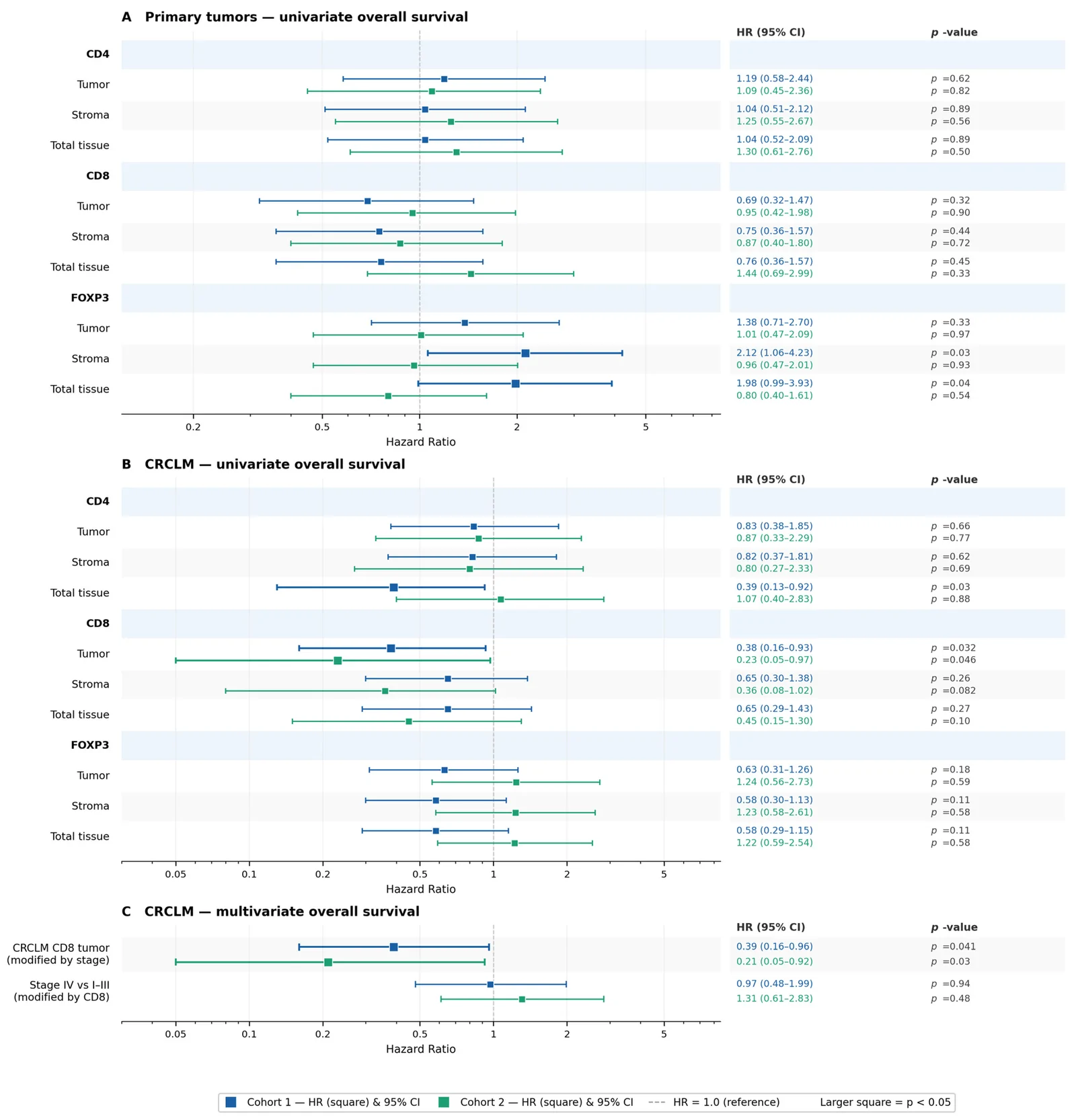
The Forest Plots of univariate analysis of TILs in (**A**) primary tumor, (**B**) CRCLM, and (**C**) multivariate analysis of stage and CD8 levels. Blue line: cohort1; green line: cohort2; square: hazard ratio; dotted line: HR = 1 (reference).

There was no significant impact on overall survival (OS) calculated from the time of diagnosis when stratified by clinicopathological characteristics except for stage at diagnosis in Cohort2 (*p* = 0.02, Table 2). The levels of CD8^+^ TILs in primary tumors showed only a minor association with improved survival that did not reach statistical significance (Cohort1 HR = 0.69 (0.32–1.47), *p* = 0.32, Cohort2 HR = 0.95 (0.42–1.98), *p* = 0.90, Figure 2A). By contrast, a significant survival benefit was seen in patients showing high CD8^+^ effector T-cell infiltration in the tumor compartment of CRCLMs in both cohorts by univariate OS analysis (Cohort1: HR = 0.38 (0.16–0.93), *p* = 0.032; Cohort2: HR = 0.23 (0.05–0.97), *p* = 0.046, Figure 1D and Figure 2B). No consistent associations were seen between the levels of CD4 or FOXP3^+^ TILs and OS in the cohorts. The survival effect of high CD8 in CRCLM remained statistically significant in multivariate Cox regression adjusted for stage (HR = 0.39 (0.16–0.96), *p* = 0.041 in Cohort1 and HR = 0.21 (0.05–0.92), *p* = 0.03 in Cohort2, Figure 2C).

Analysis of the association between TILs and clinicopathologic variables showed no significant associations of TIL levels in primary and metastatic tissues with sex, age, race, grade, and BRAF, KRAS, and MMR status. Patients with stage IV tumors at diagnosis showed significantly higher levels of CD8 in the tumor compartment of CRCLMs compared to patients with stage I–III tumors at diagnosis (*p* = 0.007 and 0.04 in Cohort1 and 2, respectively, Table 3).

**Figure 3:**
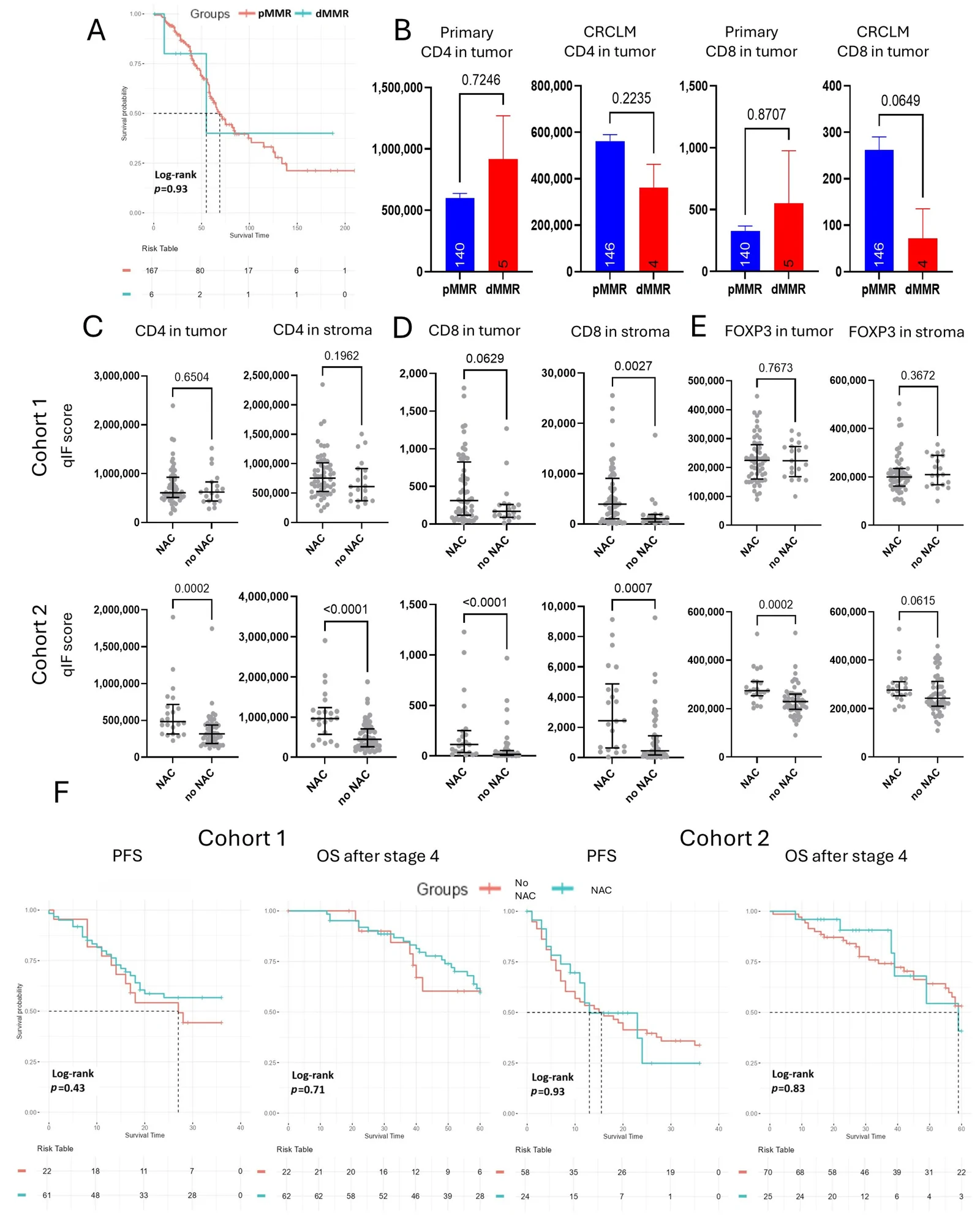
The effect of NAC on TME and survival outcomes. (**A**). Comparison of OS in patients with pMMR and dMMR tumor phenotypes; Dotted line indicates median survival (**B**). CD4^+^ and CD8^+^ TIL levels in pooled dMMR (red) and pMMR (blue) in primary and CRCLM tumor cores (**C**–**E**). CD4, CD8, and FOXP3 marker levels, respectively, in CRCLM according to neoadjuvant chemotherapy (NAC) status. Each gray dot represents the mean quantitative immunofluorescence (qIF) score of an individual patient’s CRCLM sample; *p* values shown above the line (**F**). Survival outcomes of patients stratified by neoadjuvant chemotherapy (NAC) exposure. Dotted line indicates median survival; Orange line: no NAC; blue line: NAC.

To evaluate the impact of mismatch repair status on TILs, all mismatch repair-deficient (dMMR) and -proficient (pMMR) tumors were pooled from both cohorts due to the low incidence of dMMR in these cohorts. The concordance for dMMR/pMMR status between primary colorectal tumors and CRCLMs was 100%. As expected, and despite limited statistical power to achieve definitive comparisons, primary tumors with dMMR showed numerically higher levels of CD4^+^ and CD8^+^ TIL levels than pMMR malignancies, but no significant difference in OS (Figure 3A,B). However, dMMR CRCLMs showed numerically lower CD4^+^ and CD8^+^ TIL levels than their pMMR counterparts (*p* = 0.22 and 0.06 for CD4 and CD8 in CRCLM, respectively, Figure 3B). Thus, these results suggest that T-cell infiltration is higher in dMMR primary colorectal tumors compared to pMMR primary tumors, as previously reported [23,24], but this effect was reversed in liver metastases.

To measure the impact of NAC prior to hepatectomy on TIL populations in the CRCLMs, we compared their levels in patients with and without exposure to cytotoxic therapy prior to liver resection. CD4^+^ TILs were significantly increased in patients that received NAC in all compartments of CRCLM tissues in Cohort2 (*p* = 0.0002 and *p* < 0.0001 in the tumor and stromal compartment, respectively, Figure 3C). CD8^+^ TILs were higher in NAC-treated CRCLMs from both cohorts in all compartments (*p* = 0.06 and 0.0027 in Cohort1 tumor and stroma compartments, respectively, *p* < 0.0001 and 0.0007 in Cohort2 tumor and stroma, respectively, Figure 3D). FOXP3^+^ TILs were significantly increased in NAC-treated CRCLM tumor compartments of Cohort2, although no meaningful changes were seen in Cohort1 CRCLM (*p* = 0.0002 and 0.06 in Cohort2 tumor and stroma, respectively, Figure 3E). This effect was not seen in synchronous primary tissues, except for an increase in CD4 levels in the whole tissue compartment of Cohort2 (*p* = 0.004, Supplementary Figure S3). Notably, NAC was not associated with demonstrable survival benefit in either cohort (HR= 0.75 for Cohort1 PFS, *p* = 0.43 and 0.71 for Cohort1 PFS and OS, respectively, HR = 0.97 for Cohort2 PFS, *p* = 0.93 and 0.83 for Cohort2 PFS and OS, respectively, Figure 3F).

## 4. Discussion

In this study, our results demonstrate that CD8 TILs in metastatic CRC correlate with clinicopathological characteristics and survival outcomes and suggest NAC modulates changes in the immune microenvironment, although the effects of NAC do not improve survival.

Previous studies have shown that CRCLMs correlate with shorter survival and immunotherapy resistance; for example, the combination of botensilimab and balstilimab demonstrated antitumoral activity in MSS/pMMR CRC only in cases without CRCLM [11]. A deeper understanding of the immune TME is essential for developing novel therapeutic strategies. Our findings underscore the critical role of CD8^+^ TILs in shaping the tumor immune response and influencing survival outcomes.

Our results demonstrate that the colorectal cancer TME differs between primary and metastatic tumor sites. Tregs and CD8^+^ TILs show the most pronounced inter-tumor variation, with higher levels of Tregs in primary tumors compared to CRCLMs. Previous studies on populations without CRC have shown high numbers of resident Tregs across the whole GI tract, especially in the colon and cecum [25], which might explain the higher levels of Tregs seen in primary tumors of our study. The CD8^+^ TIL population was significantly higher in the liver compared to the primary site, which supports a more prominent anti-tumor adaptive immune response in the liver. This could be due, at least in part, to the reduced presence of Tregs that are known to be able to locally suppress effector T-cell proliferation and expansion after antigen-mediated activation. Previous studies of matched primary and CRCLM tissues have shown significantly higher rates of exhausted TILs in the liver, which might be the reason why the immune response observed in our study is not effective against the CRCLM [26]. The functional state of the CD8^+^ TILs in CRCLM needs to be further investigated in protein level.

While the distinctive immune architecture and immunosuppressive milieu of the liver have been previously described [15,27], our study is the first to systematically analyze TILs quantitatively and in multiple independent patient-matched primary-CRCLM cohorts. We looked at different cutpoints for marker levels at different percentiles to optimize the outcomes of the analysis by utilizing the most meaningful percentile. With this strategy, we made sure to have the most biologically relevant results. Our findings support the work of others that have described the liver may facilitate immune escape through its inherently tolerogenic microenvironment [9,28]. For example, in immunotherapy-sensitive tumors such as melanoma and NSCLC, liver metastases are associated with poorer outcomes to immune checkpoint inhibitors, although responses can still occur in the presence of liver metastases [29]. In contrast, pMMR/MSS CRC tumors are highly unlikely to respond at any metastatic site to immunotherapy when active liver metastasis are present [30]. This is hypothesized to be due to a systemic immunosuppressive influence of liver metastases, dampening immune responses at distant sites through unknown mechanisms, including TIL exhaustion [31,32]. Additional studies have also highlighted the potential role of other immune cell populations such as macrophages in shaping the liver TME in CRCLM, which we plan evaluating in future studies [31,33]. Studies have shown that macrophage-related cytokines, including IL4, IL6, and IL8, can contribute to the broader immunosuppressive microenvironment and lower CD8 recruitment in the liver [34,35].

We identified survival benefits from CD8^+^ T-cell infiltration in tumor and stromal compartments of CRCLMs. This data is consistent with the existing literature showing a marked prognostic effect of CD8^+^ TILs in patients with colorectal cancer [36,37]. Notably, our results support a larger effect of effector TILs in metastatic lesions than in primary tumors, supporting that enhancing anti-tumoral immune response in CRCLM could influence patient outcomes. The association of immune cell infiltration improving patient outcomes is robust across multiple studies and is independent of tumor stage, mismatch repair (MMR) status, and other molecular features [38,39]. The prognostic value is particularly strong when CD8^+^ T-cells are present at high density in both the tumor core and the invasive margin, and this effect is most pronounced in higher-risk tumors, such as stage III or pT4/N1-2 disease [40,41]. Although the same effect was seen in primary tumors, it did not reach statistical significance in our study. We hypothesize that this apparent difference could be related with the fact that our cohorts included exclusively metastatic patients and the primary tumor analysis from our study cannot be directly compared to assumptions of the TME from primary tumors in cohorts of patients stage I–III as reported [42]. Differences in the representation of CD8^+^ TILs using TMA format in primary tumors could also be a contributing factor.

NAC with FOLFOX regimen is widely used to treat patients with CRCLM who have unresectable but potentially convertible CRCLMs [43]. Studies have shown improved PFS with NAC but no OS benefits [43,44]. It has been shown that NAC can increase TILs recruitment at the tumor site [45]. Our results show that although NAC increased the levels of CD8^+^ effector TILs, it did not translate into improved OS or PFS. This suggests that while existing systemic therapies may enhance immune infiltration in CRCLM, they may negatively affect their anti-tumor function. We saw an increase in FOXP3 TIL infiltration with NAC as well; although not statistically significant, this can explain to some extent why the observed immune recruitment was not effective against CRCLM. Additional novel immunostimulatory therapies or rational combinations may be able to overcome the immune resistance of CRCLM and complement the effect of NAC. Furthermore, our results suggest that while dMMR tumors exhibited a more inflamed TME in the primary site, this effect was inverted in the corresponding liver metastases, which has not been previously reported. This further suggests that despite the expected immunogenicity of dMMR tumors, when CRCLM develops this immunogenicity can be compromised by the immunosuppressive liver tissue composition and function.

Our findings of increased TILs in CRCLM from patients who received NAC support an impact on the TME by cytotoxic chemotherapy. While NAC appears to enhance local inflammatory responses in the TME and promote recruitment of T-cells, these changes appear insufficient to drive effective tumor rejection and lead to favorable outcomes [46]. Our findings align with earlier reports on the impact of NAC on the immune TME of CRCLM, which describe increased infiltration of CD8^+^ and CD4^+^ T-cells as well as macrophages in the liver following treatment [12,44,47]. We plan to investigate T-cell exhaustion markers in the future to gain a more comprehensive picture of the immune landscape in CRCLM.

We did not observe global survival benefits with NAC prior to hepatectomy by either PFS or OS analysis. This finding could be affected by patient selection bias with preferential inclusion of individuals with more advanced disease (e.g., with CRCLM) and worse prognosis who are more likely to receive NAC [43,48].

There are several limitations of our study, including its retrospective design and single-institution sample collection. By utilizing discovery and validation cohorts, we tried to mitigate this possible concern. Moreover, the small number of dMMR cases in the cohorts limits subgroup analysis and the associated conclusions. The use of TMAs could over or underrepresent the markers in the tumor samples. We addressed this issue by including multiple cores of each tumor on our TMAs to increase their representation and minimize the possible impact of intratumor heterogeneity. The consistent results obtained in independent cohorts also support the validity or our approach. Another limitation would be the use of a single technique (quantitative immunofluorescence) to evaluate the tissue immune microenvironment. Future work using higher-dimensional spatial platforms such as spatial transcriptomics or imaging mass cytometry will be required to provide deeper insights into the TME architecture and cell–cell interactions in CRCLM.

## 5. Conclusions

Our data supports the prognostic significance of CD8^+^ TILs in CRCLM and indicates that immune profiles are altered by the disease location and treatment. These findings have biomarker implications for immunotherapy response prediction and underscore the need for spatially resolved multi-site immune profiling in metastatic CRC. Additional and ongoing characterization of the activation state of immune cells in the CRCLM TME are needed to develop novel treatment strategies to overcome both the local and systemic immune resistance seen in metastatic colorectal cancer.

## Figures and Tables

**Table 1:** The clinicopathological characteristics of patients included in Cohort1 and Cohort2, values compared with χ^2^ test.

		Cohort1	Cohort2	*p* Value
Sex	Male	48 (58%)	50 (53%)	0.44
	Female	35 (42%)	45 (47%)	
Age [mean (range)]		57.65 (27–83)	57.13 (30–83)	0.99
Race	White	57 (67%)	72 (75%)	0.23
	Non-white	27 (33%)	23 (25%)	
Microsatellite stability status	pMMR	80 (98%)	88 (95%)	0.13
	dMMR	1 (2%)	5 (5%)	
Anatomical site	Left	53 (63%)	70 (73%)	0.12
	Right	31 (37%)	25 (27%)	
Histological grade	Low-intermediate	59 (71%)	57 (76%)	0.12
	High	23 (29%)	18 (24%)	
Stage at initial diagnosis	I	3 (3%)	0	0.17
	II	2 (2%)	6 (6%)	
	III	23 (27%)	27 (29%)	
	IV	55 (66%)	62 (65%)	
Received neoadjuvant therapy before metastatectomy?	yes	63 (75%)	57 (61%)	0.06
	No	21 (25%)	35 (39%)	
Duration of follow-up (mean in months)		61.25	63.87	0.67

**Table 2:** Univariate Cox proportional hazards model of sex, stage, sidedness, and mutation status.

	Univariate Overall Survival Analysis [HR (95%CI) (*p* Value)]	
	Cohort1	Cohort2
Sex (male vs. female)	1.15 (0.63–1.62) (*p* = 0.58)	0.95 (0.52–1.76) (*p* = 0.85)
Sidedness (left vs. right)	1.03 (0.51–2.24) (*p* = 0.95)	0.71 (0.38–1.35) (*p* = 0.29)
Stage at diagnosis (Stage IV vs. I–III)	1.19 (0.62–2.41) (*p* = 0.27)	1.73 (1.05–3.01) (*p* = 0.02)
BRAF mutation (V600E Mutated vs. non-mutated)	2.21 (0.66–7.39) (*p* = 0.24)	4.25 (0.90–20.1) (*p* = 0.11)
KRAS mutation (Mutated vs. non-mutated)	1.25 (0.64–2.45) (*p* = 0.50)	1.79 (0.83–3.87) (*p* = 0.13)

**Table 3:** The association of clinicopathologic characteristics in Cohort1 and 2 and marker levels in CRCLM tumor compartment, values compared with χ^2^ test.

		Cohort1									Cohort2								
		CD4 Low	CD4 High	*p* Value	CD8 Low	CD8 High	*p* Value	FOXP3 Low	FOXP3 High	*p* Value	CD4 Low	CD4 High	*p* Value	CD8 Low	CD8 High	*p* Value	FOXP3 Low	FOXP3 High	*p* Value
Age	>50	41 (74%)	14 (25%)	0.59	42 (76%)	13 (24%)	0.59	29 (52%)	26 (48%)	0.91	37 (68%)	17 (32%)	0.77	36 (66%)	18 (33%)	0.66	27 (50%)	27 (50%)	0.79
	≤50	11 (55%)	9 (45%)		10 (50%)	10 (50%)		9 (45%)	11 (55%)		15 (72%)	6 (28%)		16 (76%)	5 (24%)		11 (53%)	10 (47%)	
Sex	Female	23 (68%)	11 (32%)	0.97	25 (73%)	9 (27%)	0.64	18 (52%)	16 (48%)	0.89	27 (75%)	9 (25%)	0.44	24 (66%)	12 (33%)	0.81	22 (61%)	14 (39%)	0.13
	Male	29 (71%)	12 (29%)		27 (66%)	14 (34%)		20 (48%)	21 (52%)		25 (64%)	14 (36%)		28 (71%)	11 (29%)		16 (41%)	23 (59%)	
Side	Right	15 (68%)	7 (32%)	0.64	18 (81%)	4 (19%)	0.21	10 (45%)	12 (55%)	0.55	13 (56%)	10 (44%)	0.18	17 (73%)	6 (27%)	0.76	9 (39%)	14 (61%)	0.28
	Left	37 (70%)	16 (30%)		34 (65%)	19 (35%)		28 (52%)	25 (48%)		39 (75%)	13 (25%)		35 (68%)	17 (32%)		29 (55%)	23 (45%)	
Grade	Low-intermediate	35 (64.8%)	29 (35.2%)	0.19	38 (70.4%)	16 (29.6%)	1	26 (48.1%)	28 (51.9%)	0.64	30 (62.5%)	18 (37.5%)	0.24	33 (68.5%)	15 (31.2%)	0.50	24 (50%)	24 (50%)	0.67
	High	16 (84.2%)	3 (15.8%)		14 (73.7%)	5 (26.3%)		11 (57.9%)	8 (42.1%)		11 (84.6%)	2 (15.4%)		7 (53.8%)	6 (46.2%)		5 (38.5%)	8 (61.5%)	
Stage	I-III	24 (85.7%)	4 (14.3%)	0.04	25 (89.3%)	3 (10.7%)	0.007	17 (60.7%)	11 (39.3%)	0.30	22 (81.5%)	5 (18.5%)	0.14	23 (85.2%)	4 (14.8%)	0.04	20 (74.1%)	7 (25.9%)	0.005
	IV	28 (60.9%)	18 (39.1%)		26 (56.5%)	20 (43.5%)		21 (45.7%)	25 (54.3%)		30 (62.5%)	18 (37.5%)		29 (60.4%)	19 (39.6%)		18 (37.5%)	30 (62.5%)	
Recurrence after hepatectomy	No	24 (67%)	12 (33%)	0.73	22 (61%)	14 (39%)	0.17	18 (50%)	18 (50%)	1	17 (68%)	8 (32%)	1	15 (60%)	10 (40%)	0.41	10 (40%)	15 (60%)	0.58
	Yes	27 (73%)	10 (27%)		29 (78%)	8 (22%)		19 (51%)	18 (49%)		30 (68%)	14 (32%)		32 (73%)	12 (27%)		22 (50%)	22 (50%)	
MMR status	pMMR										50 (70.4%)	21 (29.6%)	0.76	49 (69%)	22 (31%)	1	36 (50.7%)	35 (49.3%)	1
	dMMR										2 (50%)	2 (50%)		3 (75%)	1 (25%)		2 (50%)	2 (50%)	
KRAS/NRAS	Wild type	24 (70.6%)	10 (29.4%)	1	21 (61.8%)	13 (38.2%)	0.39	17 (50%)	17 (50%)	1	22 (62.9%)	13 (37.1%)	0.50	23 (65.7%)	12 (34.3%)	0.90	13 (37.1%)	22 (62.9%)	0.23
	Mutated	24 (68.6%)	11 (31.4%)		26 (74.3%)	9 (25.7%)		17 (48.6%)	18 (51.4%)		20 (74.1%)	7 (25.9%)		19 (70.4%)	8 (29.6%)		15 (55.6%)	12 (44.4%)	
BRAF V600E	Wild type	39 (68.4%)	18 (31.6%)	1	38 (66.7%)	19 (33.3%)	0.29	27 (47.4%)	30 (52.6%)	0.35	31 (63.3%)	18 (36.7%)	1	31 (63.3%)	18 (36.7%)	1	19 (38.3%)	30 (61.2%)	1
	Mutated	3 (75%)	1 (25%)		4 (100%)	0		3 (75%)	1 (25%)		2 (66.7%)	1 (33.3%)		2 (66.7%)	1 (33.3%)		1 (33.2%)	2 (66.7%)	

## Data Availability

The data supporting the findings of this study are available from the corresponding author upon reasonable request.

## Supplementary Materials

The following supporting information can be downloaded at: https://www.mdpi.com/article/10.3390/cancers18183042/s1, Figure S1. Representative example of tissue segmentation in Cohort2. Overlay of the automated tissue segmentation mask on the original image (green tumor; blue stroma); Figure S2. TIL distribution and correlations A. The QIF scores of TILs in Cohort1 and Cohort2, TIL levels in tissue compartments shown on the right, CD4 in yellow, FOXP3 in black, CD8 in red B. Pairwise comparison and correlations of TIL markers. Each dot represents a tissue core marker level on TMA; Figure S3. The QIF scores of synchronous primary tumors after receiving NAC. Each gray dot represents the mean quantitative immunofluorescence (qIF) score of an individual patient’s primary tumor sample.
