# Supplementary material for: Clinical Significance of Tumor Infiltrating Lymphocytes and Modulation by Neoadjuvant Chemotherapy in Colorectal Cancer Liver Metastases
Source: Cancers (Basel). 2026 Sep 19;18(18):3042. doi: 10.3390/cancers18183042 (PMC13604633; doi:10.3390/cancers18183042)
Supplement: Supplementary file 1 [file cancers-18-03042-s001.zip › cancers-4536954-supplementary.pdf]

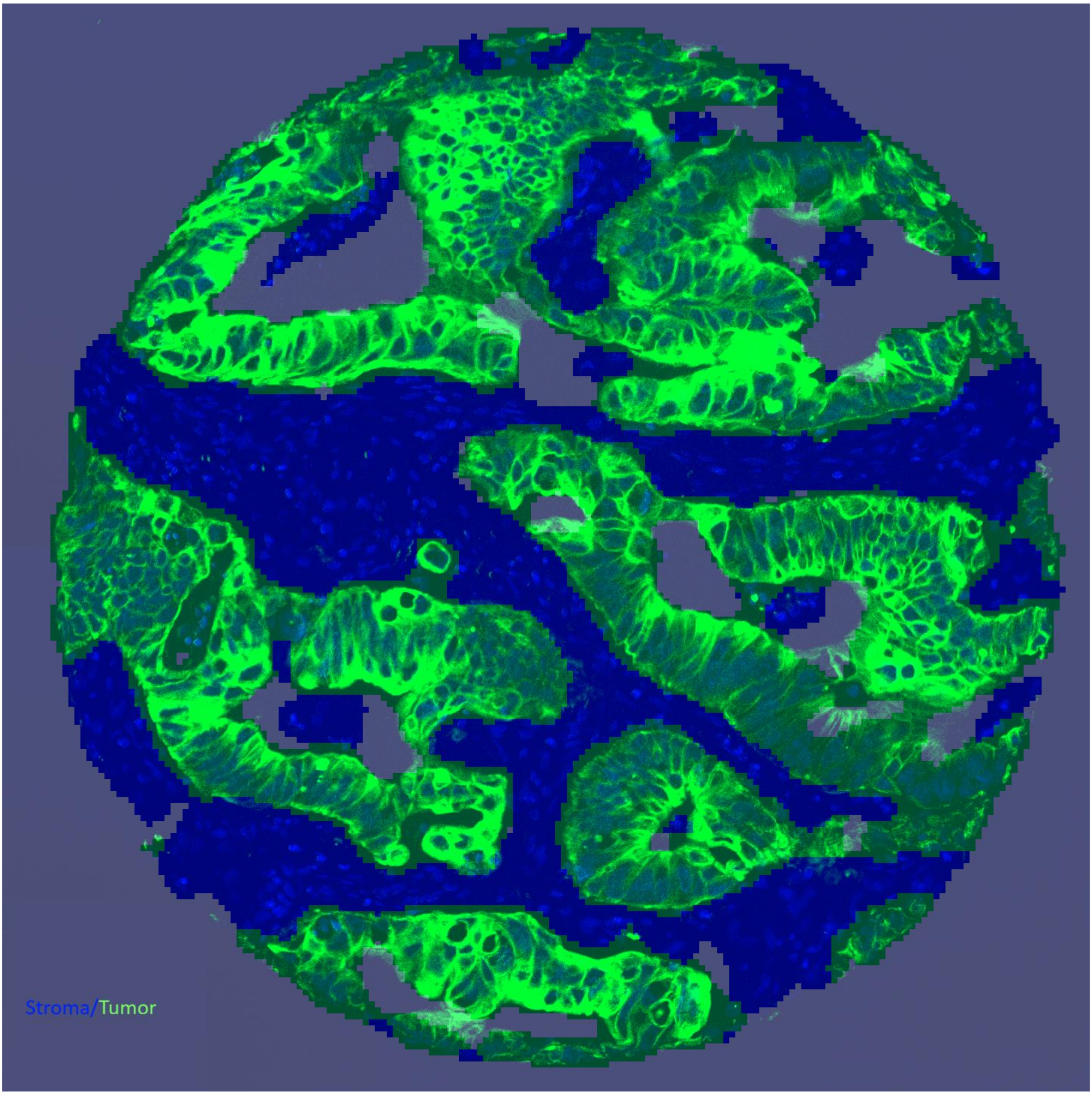

Figure S1. Representative example of tissue segmentation in Cohort2. Overlay of the automated tissue segmentation mask on the original image (green: tumor; blue: stroma).

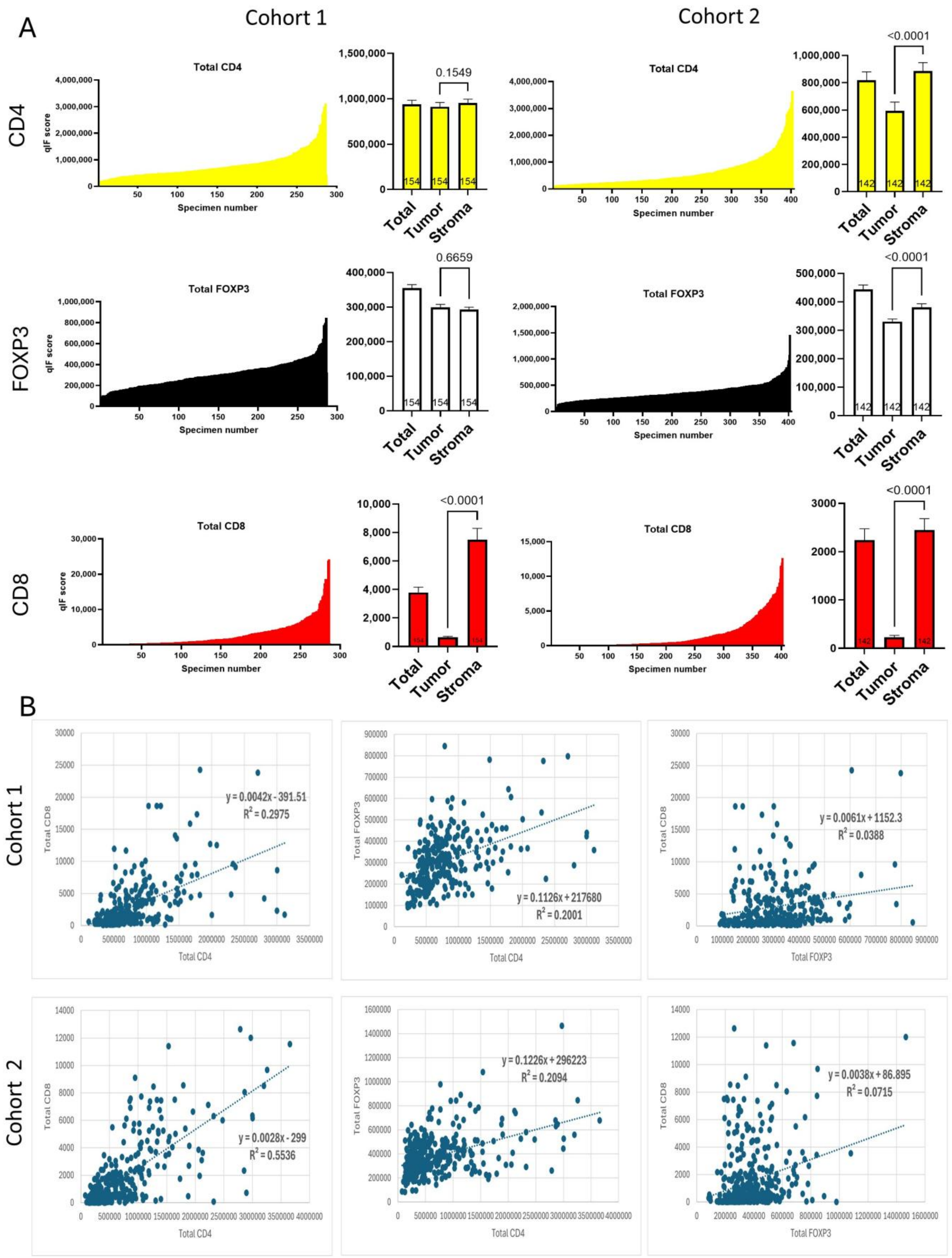

A
Cohort 1
Cohort 2
CD4
FOXP3
CD8
Total CD4
Total FOXP3
Total CD8
qIF score
Specimen number
Total
Tumor
Stroma
0.1549
0.6659
<0.0001
154
142
B
Cohort 1
Cohort 2
Total CD4
Total CD8
Total FOXP3
y = 0.0042x - 391.51
R² = 0.2975
y = 0.1126x + 217680
R² = 0.2001
y = 0.0061x + 1152.3
R² = 0.0388
y = 0.0028x - 299
R² = 0.5536
y = 0.1226x + 296223
R² = 0.2094
y = 0.0038x + 86.895
R² = 0.0715

Figure S2. TIL distribution and correlations A. The QIF scores of TILs in Cohort1 and Cohort2, TIL levels in tissue compartments shown on the right, CD4 in yellow, FOXP3 in black, CD8 in red **B.** Pairwise comparison and correlations of TIL markers. Each dot represents a tissue core marker level on TMA.

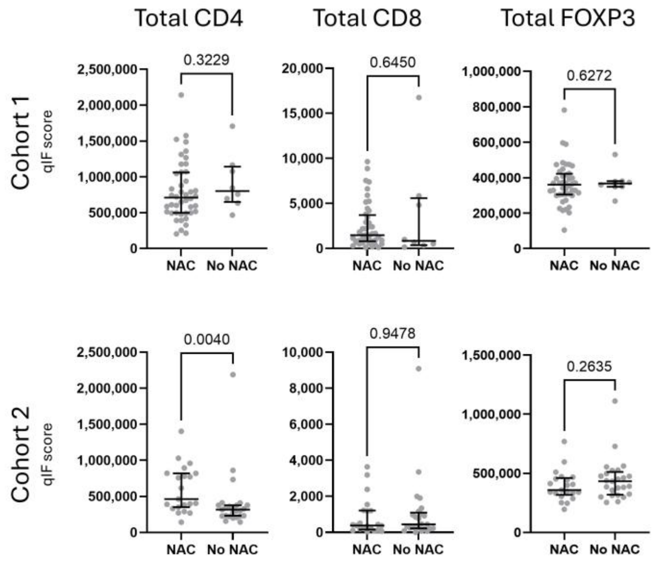

Figure S3. The QIF scores of synchronous primary tumors after receiving NAC. Each gray dot represents the mean quantitative immunofluorescence (qIF) score of an individual patient's primary tumor sample.
